# New Photodegradation Products of the Fungicide Fluopyram: Structural Elucidation and Mechanism Identification

**DOI:** 10.3390/molecules23112940

**Published:** 2018-11-10

**Authors:** Tessema F. Mekonnen, Ulrich Panne, Matthias Koch

**Affiliations:** 1 Department of Analytical Chemistry and Reference Materials, Bundesanstalt für Materialforschung und -prüfung (BAM), Richard-Willstätter Str. 11, 12489 Berlin, Germany; tessema-fenta.mekonnen@bam.de (T.F.M.); ulrich.panne@bam.de (U.P.); 2 School of Analytical Sciences Adlershof (SALSA), Humboldt-Universität zu Berlin, Unter den Linden 6, 10099 Berlin, Germany

**Keywords:** photodegradation, transformation products, LC-MS/MS, HRMS, fungicide

## Abstract

Identifying the fate of agrochemicals is important to understand their potential risk for living organisms. We report here new photodegradation products (PPs) of the fungicide fluopyram. The PPs were produced by irradiating a fluopyram standard in 0.1% acetonitrile aqueous media by a 150-W medium pressure Hg-lamp that emits wavelengths between 200–280 nm. The structural elucidation of PPs was achieved by combining the retention time, isotopic pattern, targeted fragmentation, and accurate mass measurements using liquid chromatography-tandem mass spectrometry (LC-MS/MS) and high resolution-MS (HRMS). In addition to previously known PPs, seven new PPs of fluopyram were identified in this work: mainly dihydroxyl and hydroxylimide fluopyram as well as mono, di, and trihydroxyl lactam. Additionally, two PPs were found to be formed by rearrangement after the loss of H_2_C=CH_2_. Hence, the results of the work contribute to extending the current knowledge regarding the photoinduced fate of agrochemicals, and fluopyram in particular.

## 1. Introduction

Pesticide residues such as fungicides, insecticides, and herbicides are major threats to food safety and consumers’ health. They are able to enter living organisms either by direct contact (inhalation, or dermal) or indirectly through food webs [1]. The latter is the major exposome pathway [2]. Due to their chronic and acute toxicity, pesticides are one of the most monitored residues throughout the world. However, their consumption is almost inevitable. On the other hand, they undergo numerous biotic (e.g., metabolism in living organisms and microbial system) and abiotic (e.g., food processing, waste treatment, and photochemical reactions) transformation processes [3,4,5]. Multiple stresses in biota or environmental compartments enhance the transformation of the parent residue to a new product, which might be more toxic than the parent compound [6,7]. Depending on their physicochemical properties, some of these residues might persist for several years, bioaccumulate in living organisms, or cause immediate fatal damages to the overall ecosystem [8,9,10]. However, monitoring the transformation products of pesticides is one of the greatest analytical challenges from two perspectives. The first challenge is the shortage of commercially available authentic transformation product standards [11]. Secondly, different transformation sources cause different degradation processes and types of transformation products. Meanwhile, their occurrence in different foodstuff is of concern [3,12,13]. Thus, this work primarily aims to elucidate the photodegradation products (PPs) of a fungicide fluopyram.

Fluopyram (FLP), (*N*-[2-[3-chloro-5-(trifluoromethyl)-2-pyridinyl]ethyl]-2-(trifluoromethyl) benzamide), (electronic supplementary material (ESM), Figure S1), is relatively new succinate dehydrogenase inhibitor (SDHI) fungicide that is used to protect different fruits from fungi damage. Mekonnen et al. [14] investigated the metabolic transformation processes of FLP by using an electrochemical flow-through cell in comparison to human and liver microsome assays. In this study, extensive oxidative metabolites of FLP by hydroxylation, imine formation, epoxidation, *N*-dealkylation, and the nucleophilic substitution of chlorine were reported. However, the possible fate of FLP in environmental processes (e.g., photolysis/photochemical, advanced oxidation processes, and chlorination) are not well studied yet. Nevertheless, Dong and Hu [15] reported lactam FLP, dechlorinated FLP, and hydroxyl substitution of chlorine as the three major PPs of FLP. However, to the best of the authors’ knowledge, there is no other existing data on the photodegradation mechanisms of FLP.

We report here new PPs of FLP in aqueous media by direct photolysis using a medium pressure mercury lamp that emits UV-C light. Products were structurally elucidated by LC-MS/MS and accurate mass measurements using HRMS. Seven new PPs, namely mono, di, and trihydroxyl lactam, dihydroxyl FLP, imide FLP, and two products by losing H_2_C=CH_2_ have been identified/elucidated in this work. This is the first work that reports the photodegradation of FLP by hydroxylation and rearrangement mechanisms.

## 2. Results and Discussion

This investigation sought the structural and mechanism elucidation of new PPs of FLP. After irradiation with a medium-pressure Hg-lamp (λ = 200–280 nm, 150 W) in aqueous media, new PPs were identified. The hydroxylation of parent FLP and lactam FLP were newly identified PPs by the outlined experiment. Structural elucidation was performed by combining the information obtained from LC-MS/MS and HRMS. The first insights of the molecular ions of the PPs were obtained by scanning in the first quadrupole of MS/MS in comparison to the respective blank and control samples (without UV-irradiation). The *m*/*z* traces were mapped based on the retention time to have insights about possible isotopic patterns or adducts that elute at the same retention time (and could be from the same PP). Further structural elucidation was obtained by the data-dependent fragmentation of each PP (on MS/MS and MS^3^). The final structural assignment was performed based on an accurate mass and formula determination using high-resolution mass spectrometry (HRMS) by taking a deviation of ≤5 ppm as a threshold limit.

### 2.1. Photodegradation Properties of FLP and Its PPs

**Figure 1:**
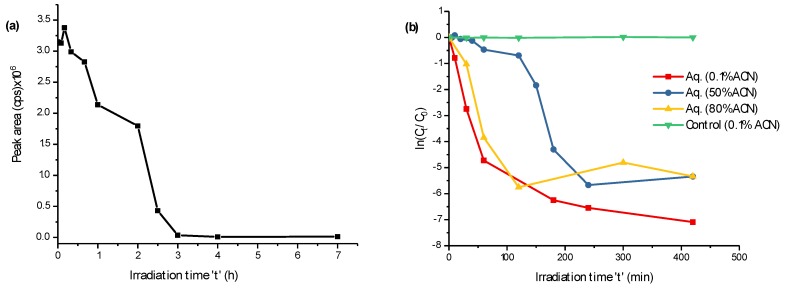
Degradation of fluopyram (FLP) (0.1 mmol L^−1^) vs. time of irradiation at λ = 200–280 nm (**a**) and FLP degradation kinetics with varying acetonitrile (ACN) compositions in aqueous solution (**b**). The *y*-axis in (**b**), ln(C_t_/C_0_), represents the natural logarithm of FLP concentration ratio after being irradiated for time ‘t’ (C_t_), before irradiated (C_0_).

More than 90% of FLP was photodegraded after irradiation by UV-C for three hours (Figure 1a), but the degradation seems not completed yet after seven hours of irradiation (Figure 1b). Since the degradation mechanism could vary by the chemical composition of the standard solution, the degradation kinetics were investigated in aqueous media with 0.1%, 50%, and 80% of acetonitrile (ACN) without a photocatalyst in parallel to the control (Figure 1b). In all of the cases, FLP degraded well, with a relatively fast degradation in 0.1% of ACN. The control is the same amount of FLP in a 0.1% ACN aqueous solution in a colorless glass container exposed to natural sunlight (for the same irradiation time). As evidenced in Figure 1b, the FLP degraded exclusively by UV-C and was stable under sunlight irradiation. After scanning the blank, control, and UV-C irradiated samples on the first quadrupole (+Q1) of MS/MS, many *m*/*z* appeared to be as a PP. To discriminate possible adducts (+Na, +K, +NH_4_^+^) and isotopic peaks (mainly from the C_3_-Cl isotope on the pyridine ring), the *m*/*z* traces were separated by LC-MS/MS, and those elutes at different retention time were considered as suspected PPs.

A previous report shows the photolysis of FLP to lactam in aqueous media by sunlight via cyclization between C_3_-Cl of the pyridinyl and C_16_-H of the benzamide aromatic rings by losing a neutral HCl [16]. However, in our case, FLP was stable after being exposed to natural sunlight for two weeks. Furthermore, Dong and Hu reported Cl- substitution by the HO• and H• of FLP after exposing at ≥200 nm wavelength in the presence of photocatalysts [15]. In both reports, the known photoinitiator (the chloride site), is highly susceptible to UV irradiation and produces a hydroxylated and/or protonated substitution of –Cl. However, the final products could be greatly varied depending on the irradiation exposure time, chemical composition, and energy of UV. Meanwhile, many industrial treatments use UV-C (germicidal UV light) for effective treatment. Hence, UV-C (200–280 nm wavelength) was selected to investigate the PPs of the intended compound.

**Figure 2:**
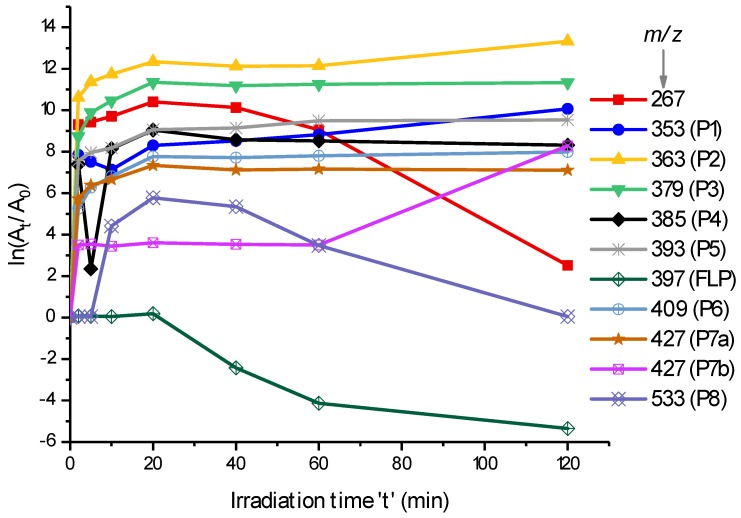
FLP degradation and photodegradation products’ (PPs) formation kinetics in 0.1% ACN aqueous solution during irradiation (λ = 200–280 nm). The *y*-axis, ln(A_t_/A_0_), represents the natural logarithm of the peak area ratios of each PP after irradiated for time ‘t’ (A_t_) to before irradiated (A_0_) by considering a constant unity at t = 0.

Although there are many peaks that were resolved by retention time, the *m*/*z* 363 (P2), 379 (P3), 393 (P5), 409 (P6), 427 (P7), 429 (P9), 361 (P10), and 377 (P11), which were formed by the hydroxylation of FLP or lactam, were identified in this work. Additionally, *m*/*z* 351 (P1) and 385 (P4) were identified as rearrangement reaction products, and *m*/*z* 533 was identified as a dimer formation. The kinetics of some of these *m*/*z* at different irradiation times are shown in Figure 2 as ln(A_t_/A_0_), where A_t_ and A_0_ are the peak areas after irradiation for time ‘t’ and before irradiation, respectively. Since A_0_ is zero for the PPs, a constant unity is taken to simplify the calculation.

As shown in Figure 2, most of the products were formed in the first 20 min and obtained a slight increase in the following irradiation times except for *m*/*z* 267 and 533, which decrease tremendously after 20 min. Hence, the further degradation of FLP could produce other products that are not addressed yet. Obviously, some of the products could be interchangeable i.e., the products could be produced from another PP rather than the parent compound. Inline of this, P1 and P2 are increased after 20 min and P7b is increased after 60 min. The highest formation kinetics of P2 and P3 could confirm many portions of FLP that are degraded through Cl-substitution rather than other mechanisms. On the other hand, P1 and P4 (both by rearrangement reactions) decrease for around 10 min, and then increased. This could confirm that the two products follow the same formation mechanism.

### 2.2. Identification of Dechlorinated PPs

**Figure 3:**
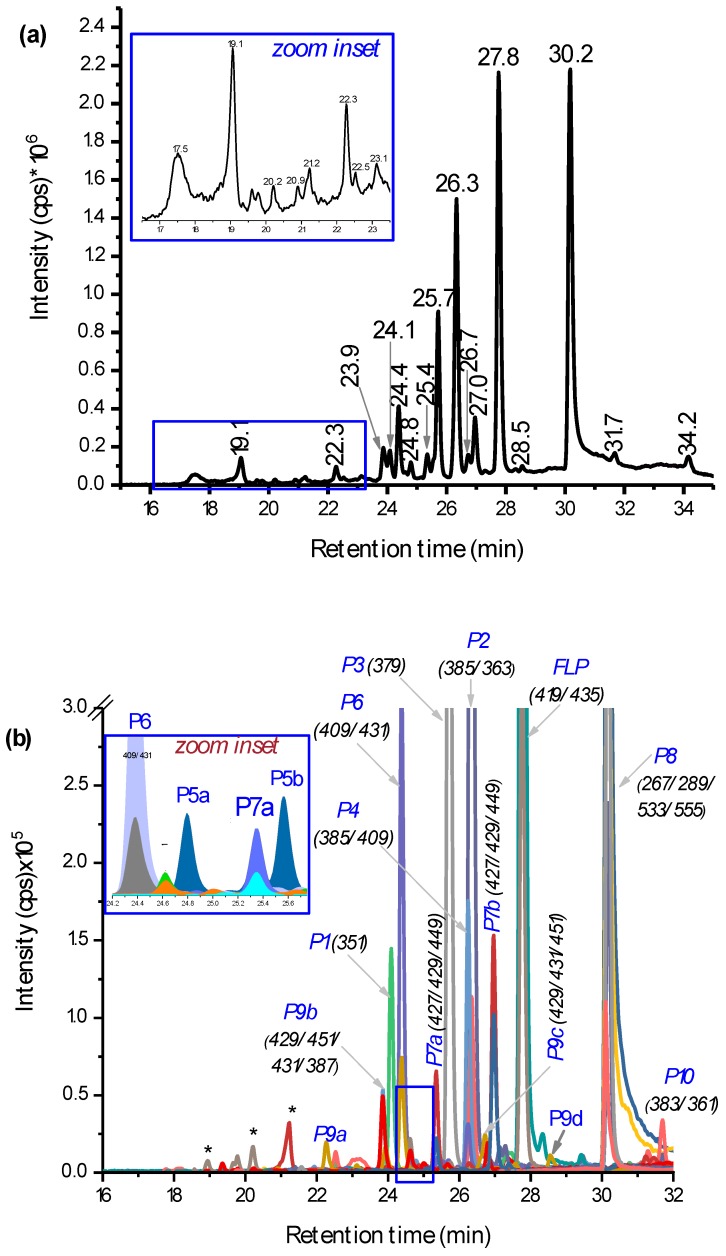
Total ion chromatogram (TIC) (**a**) and extracted ion chromatogram (EIC) (**b**) of PPs of 0.1 mmol L^−^^1^ fluopyram (FLP) measured by liquid chromatography-tandem mass spectrometry (LC-MS/MS) after being irradiated for 2 h with germicidal UV light (UV-C) light (150 W, λ = 200–280 nm). The peaks were assigned by retention time (**a**), *m*/*z* traces eluted at the specific time (**b**), and * not identified.

Chromatographic separation was performed by LC-MS/MS on a full scan within 100–1000 *m*/*z* (total ion chromatogram (TIC) in Figure 3a) and by selected ion monitoring on positive electrospray ionization, (+)ESI (Figure 3b). The extracted ion chromatogram (EIC) in Figure 3b shows many characteristic peaks that were helpful to identify the structures of the possible PPs. Especially, the presence of Cl-isotopes and +Na- or +K-adducts were used in addition to the fragmentation and HRMS data. The three known PPs reported by Dong and Hu [15] were eluted at 25.7 min, 26.3 min, and 31.7 min (Figure 3a) for Cl-substitution by H• (P2 in Figure 3b), Cl-substitution by HO• (P3), and lactam FLP (P10), respectively. Their structural assignment could be identified easily from a lack of Cl-isotopes in their MS/MS spectra (see ESM Figure S2a,b for spectra and possible fragmentation patterns). Additionally, on HRMS, their accurate masses were measured with +2.7 ppm, 0.0 ppm, and −0.2 ppm deviation from the theoretical masses (Table 1). As further evidence, the elution order on a reversed phase analytical column as P3, P2, and P10 agrees with their polarity profiles. The peak at 27.8 min is non-degraded FLP ([M + H]^+^ at *m*/*z* 397, +Na at *m*/*z* 419, and +K at *m*/*z* 435). Meanwhile, the focus of this study was to identify other new PPs. As evidenced in both Figure 2 and Figure 3a,b, many other peaks were well separated and characterized by *m*/*z* eluted at the same retention time.

### 2.3. LC-MS/MS Analysis of Hydroxyl FLP PPs

In addition to the known dechlorinated PPs (P2, P3, and P10), hydroxylation of the parent FLP was found as the main PP. Different isoforms of dihydroxyl FLP (*m*/*z* 429: P9) were produced by UV-C irradiation, such as for instance, the peaks at 22.3 min and 26.7 min (Figure 3a) showing P9, including its Cl-isotope at *m*/*z* 431 and +Na-adduct at *m*/*z* 451/453 (Figure 3b). The fragmentation pattern on QTRAP-MS/MS shows peaks at *m*/*z* 411 (−H_2_O), 397, 240, 222, 173, 145, 131, and 115 (Table 1 and ESM Figure S3a). The product ions at *m*/*z* 173 and 145 confirm the presence of the trifluoromethyl benzamide ring, and *m*/*z* 397 confirms the structure of the FLP. Other fragment ion traces were also comparable to the products reported by Mekonnen et al. using electrochemical or liver microsomes [14]. Furthermore, it was confirmed by HRMS measurement with a 1.4 ppm mass error from the theoretical value (Table 1). However, monohydroxylated FLP was not detected, which could be explained by high concentrations of HO• produced by UV-C leading to the fast hydroxylation of all of the possible sites. Since the experiment was performed in aqueous media, a higher concentration of reactive oxygen species such as HO^−^ or HO• is expected. Beside this, it seems that different isomeric dihydroxyl FLP were produced when FLP is irradiated for a longer time. In total, four dihydroxyl isoforms at 22.3 min, 24.1 min, 26.7 min, and 28.5 min (P9a–d in ESM Figure S6b) were found, but no monohydroxylated compound was found. Nevertheless, some of the dihydroxylated products could be also *N*-oxides or hydroxylation at the aromatic ring, which gave the same *m*/*z*. The possible isomeric peaks were not further elucidated, since it requires fractionation by preparative high-performance LC (HPLC) and NMR investigation.

**Figure 4:**
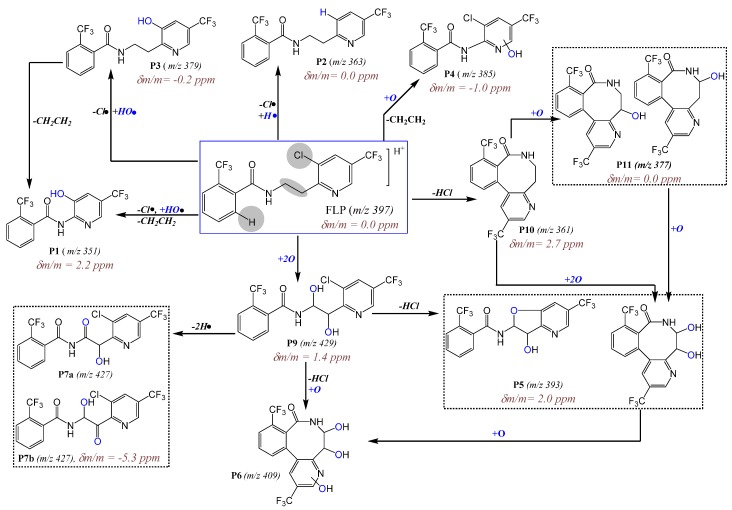
Photodegradation mechanism of FLP in 0.1% ACN aqueous media after irradiated for 2 h by Hg-lamp (150 W, λ = 200–280 nm). The circled rectangles represent PPs that could be stereoisomers (the same molecular formula but different structures).

Additionally, two isomeric peaks (P7a and P7b) with *m*/*z* 427 (2 Da lower than the dihydroxyl FLP) were eluted at 25.4 min and 27.0 min (Figure 3a). P7, its Cl-isotope at *m*/*z* 429, and +Na-adduct at *m*/*z* 449 (Figure 3b and ESM Figure S6b) all were eluted at the same retention time. Furthermore, the fragments appeared to give *m*/*z* 409 (−H_2_O), 380 (−CO), 238, 189, 173, and 145 (see ESM Figure S3b). From the formation kinetics in ESM Figure S6d, the two products P7a and P7b, suggest that the isomers could be interchanged with each other through the course of irradiation. Therefore, P7 could be an imide formed by alcohol oxidation of the dihydroxyl FLP (P9). Alcohol oxidation to a carbonyl group by UV exposure was also reported elsewhere [17,18]. The other product with *m*/*z* 427 that elutes early at 21.2 min was not confirmed by either of Cl-isotope or Na-adduct (Figure 3a and b). From the point of toxicity, the imide could be a toxic PP, since it gives reactive species that damage cellular activities [19]. In general, two groups (dihydroxyl and imide) of new hydroxyl PPs were identified (Figure 4 for structures).

### 2.4. LC-MS/MS Analysis of Hydroxyl Lactam FLP PPs

Other main photodegradation pathways were found to be the hydroxylation of lactam PP. Mono and dihydroxyl lactam (see Figure 4 for structures, P11 and P5) PPs were identified with *m*/*z* 377 (δm/m = 0.0 ppm, Table 1) and *m*/*z* 393 (δm/m = 2.0 ppm, Table 1), respectively. They eluted at 24.8 min and 25.6 min (two isomeric peaks for P5a and P5b in the zoom-inset part of Figure 3b). The MS/MS spectra of P5 appeared to be *m*/*z* 375, 176, and 188 and P11 at *m*/*z* 359 (−H_2_O), 345, 176, and 188 (ESM Figure S4a and Table 1). A lack of Cl-isotopic peak at *m*/*z* 379 and 395 and product ion with *m*/*z* 145 and 173 in both P5 and P11 confirm the presence of a lactam structure. The monohydroxylated lactam FLP (see Figure 4 for structures) could be formed at C_7_ or alternatively at C_8_-positions. Another main PP with a lactam structure was found at *m*/*z* 409, which eluted at 24.4 min (P6 Figure 3a,b). It eluted with an +Na-adduct at *m*/*z* 431 (see ESM Figure S6b) and gave product ions at *m*/*z* 391 (−H_2_O), 377 (lactam dihydroxyl), 359 (lactam), 202, 170, 131, and 115 (see ESM Figure S4b), all without Cl-isotope. Initially, it was supposed to be a hydroxyl imide product with an HO• substitution of −Cl-, but the absence of product ions at *m*/*z* 173 and 145 led to the conclusion of a trihydroxyl lactam. Further hydroxylation could occur either in the benzamide or pyridine ring. As reviewed by Burrows et al., different *N*-containing pesticides could produce *N*-oxide PPs when they were treated by UV [20].

In general, five PPs by hydroxylation reaction were identified (P5, P6, P7, P9, and P11). When aqueous solutions and molecular oxygen are irradiated by UV light, many reactive oxygen species could be formed [7]. Radicals could be formed by the direct photolysis of water as H_2_O + 𝒉ν → H• + •OH, where 𝒉 is Planck’s constant (6626 × 10^−34^ Js) and ν is frequency. Furthermore, the HO• radical could change to peroxides (HO• + HO• → H_2_O_2_). On the other hand, H• can react with dissolved O_2_ and produce different types of reactive species (H• + O_2_ → HO_2_• and HO_2_• → O_2_^•−^). Hence, these hydroxyl and superoxide radical species have in general a very short lifetime and are capable of degrading many organic molecules. Especially, the HO• could make a nucleophilic attack to produce hydroxyl products. Therefore, the hydroxylated products of FLP and lactam FLP could be formed in a similar mechanism.

The other main PPs found in this work were *m*/*z* 351, 385, and 533 (P1, P4, and P8 in Figure 3b and Figure 4). The one with *m*/*z* 385 elutes with *m*/*z* 387 (Cl-isotope) and 409 (+Na) and fragmented to *m*/*z* 198 (most abundant), 173, 162, and 145 (see ESM Figure S5a and Table 1). The fragments at *m*/*z* 173 and 145 confirm the presence of the trifluorobenzamide group in the structure, and the most abundant peak at *m*/*z* 198 could be due to the HO• hydroxylation of the pyridine ring. Hence, P4 (δm/m = −1.0 ppm, Table 1) is suggested to be a rearrangement of PP by losing H_2_C=CH_2_. Due to the peak at *m*/*z* 198 (ESM Figure S5a), the hydroxylation could most probably occur at the pyridine ring. Beside this, P1 (δm/m = 2.2 ppm Table 1) was fragmented to *m*/*z* 335 (−H_2_O), 321, 173, 164, 145, 131, and 115 (see ESM Figure S5b), which assures the presence of trifluoromethyl benzamide, at least one HO•, and without −Cl-. The intense peak at *m*/*z* 164 could be a fragment of the pyridine ring with HO• substitutes of −Cl-. Meanwhile, P1 varies by one −Cl- atom from P4. Hence, all this information led to the conclusion that P1 could be formed by rearrangement reactions from P3 by losing H_2_C=CH_2_.

On the other hand, *m*/*z* 533, 555 (533+Na), 267, and 289 (267+Na) were eluted together at 30.1 min (Figure 3a,b). The fragmentation of P8 gave only product ion *m*/*z* 267 (with Cl-isotope) and 249 (see ESM Figure S5c). This could be due to the P8 being formed as a dimer of *m*/*z* 267 (2 × 267 – H = 533).

To know further about the possible structures and the correlation between *m*/*z* 267 and P8, their EIC and rate of formation are plotted in ESM Figure S6a,c. In the first 10 min of irradiation, only *m*/*z* 267 was produced. After 10 min, the peak area ratio of both *m*/*z* 267 and P8 behave similarly; peaks were produced within 10–20 min and then reduced. Production of P8 after *m*/*z* 267 could support the possibilities of dimer production. However, the structures of P8 were not identified in this study, since the fragmentation experiment could not show any characteristic peaks related to FLP. In summary, seven new PPs of the fungicide FLP (mainly hydroxylated PPs from the parent FLP and lactam) were identified. Although lactam was reported before, its mono, di, and tri-hydroxylated products were identified for the first time in this work. Depending on irradiation time, it seems that PPs with further hydroxylation on the aromatic rings were formed. 

## 3. Materials and Methods

### 3.1. Chemicals

Analytical standard of FLP (99.9% purity) was purchased from Sigma-Aldrich (Steinheim, Germany). Ammonium formate from Fluka Chemie Merck (Buchs, Switzerland) and formic acid from J.T. Baker (Arnhem, The Netherlands) were purchased in p.a. grade. An HPLC grade ACN, 99.90%) was purchased from Th. Geyer (Renningen, Germany). Ultrapure water was produced by a Seralpur PRO 90 CN system (Ransbach-Baumbach, Germany).

### 3.2. Photodegradation

**Figure 5:**
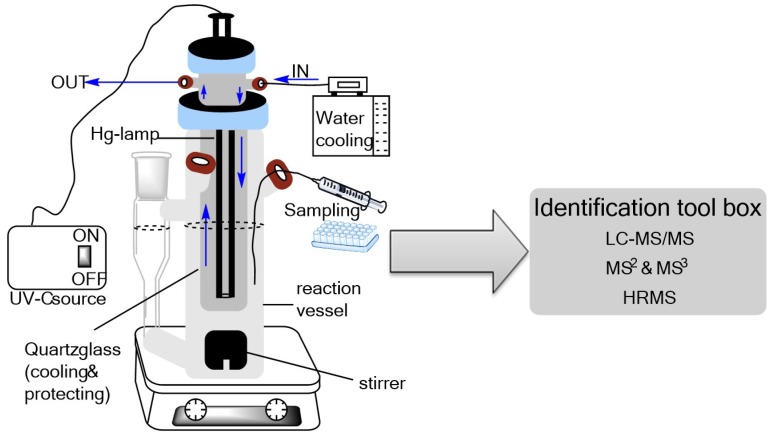
Schematic diagram of the photodegradation experimental setup.

To investigate the PPs of FLP, a UV reactor equipped with a water cooling system and a medium-pressure mercury lamp (TQ 150 W, Heraeus Noblelight GmbH, Hanau, Germany) that emitted light in the UV-C wavelength range (λ = 200–280 nm) was used (Figure 5). The measured irradiance of the UV lamp was 255 mW cm^−2^. The vessel was filled with 200 mL of 0.1 mmol L^−1^ FLP standard solution in ACN/H_2_O (0.1% ACN *v*/*v*) and stirred constantly with a magnetic stirrer (700 rpm). After cooling down the system to 12.5 °C, the UV lamp was switched on, and aliquots (1 mL) were collected after 2 min, 5 min, 10 min, 20 min, 40 min, 60 min, 120 min, 180 min, 360 min, and 420 min. The experiment was performed in triplicate.

### 3.3. LC-MS/MS Analysis of PPs

The identification and characterization of PPs were performed on an Agilent 1200 series HPLC hyphenated to an AB Sciex 4000 QTRAP^®^ MS/MS (Foster City, CA, USA). In order to obtain the structural information for each product, the MS/MS spectra of the targeted precursor ions was collected by a data-dependent scan with dynamic fill time (DFT) on (+)ESI for 3.5 min from the 2-h irradiated products by direct infusion (5 µL min^−1^). The scan rate was 4000 Da/s with 5 s of delay time and a +5000 V ion source for all of the products. An Agilent eclipse XDB C8 analytical column (150 × 2.1 mm, 5 µm) was used for separation of the PPs. Water (A) and ACN (B) both with 0.1% *v*/*v* formic acid were used as a mobile phase. The gradients were isocratic with 60% A for 8 min, linearly dropped to 0% in 2 min, kept for 5 min, and then increased to 50% A within 5 min and kept for the next 10 min; finally, it switched to its original condition for 10 min. The total analysis time was 40 min. The flow rate was 300 µL min^−1^ with a column compartment temperature of 45 °C. Blank (without analyte) and control samples (unirradiated) were analyzed at the same time.

The QTRAP-MS/MS conditions were: collision gas (CAD), 5; curtain gas (CUR), 30 psi; source gas 1 (GS1), 60 psi; source gas 2 (GS2), 20 psi; source temperature, 500 °C, and dwell time, 300 msec. For targeted fragmentation, similar conditions were used, except for 40 psi GS2, +95 eV declustering potential (DP), 45 eV collision energy (CE), 13 eV cell exit potential (CXP), and 10 eV entrance potential (EP) were fixed for all of the PPs. Data was acquired using Analyst 1.5.2 software (AB Sciex, Foster City, CA, USA).

### 3.4. Confirmation by HRMS

HRMS data were acquired by an Exactive^TM^ Orbitrap mass spectrometer (Thermo Fisher, Bremen, Germany) for the accurate mass determination of each PP. The Orbitrap was equipped with an ESI source that was operated at +5000 V spray voltage. Aliquots from two-hour irradiated samples were diluted a thousand-fold by 50% ACN containing 0.1% formic acid and infused to an ESI source by a microsyringe (5 µL min^−1^ flow rate). A full scan on (+)ESI within 120 *m*/*z* to 2000 *m*/*z* range and 100000 nominal mass resolutions (5 Hz scan rate) was performed. Data was acquired by Xcalibur^TM^ software (Thermo Fisher). Additionally, open source software MZmine-2.33 [21] was used for the further manipulation of MS data.

## 4. Conclusions

In this study, photodegradation products (PPs) of the fungicide fluopyram (FLP) were investigated. In addition to three previously known PPs, seven new transformation products of the intended compound were identified. The newly identified compounds derived from hydroxylation of the parent FLP and the secondary PPs of lactam FLP. Four isomers of dihydroxyl FLP and two isomers of hydroxylimide FLP were found from the direct photolysis of FLP. Additionally, mono, di, and trihydroxyl lactam PPs were successfully elucidated by using retention times, fragmentation patterns, isotopic peaks, and HRMS accurate mass measurements. Furthermore, two rearrangement products by the loss of a neutral H_2_C=CH_2_ from FLP were identified either with additional hydroxylation as well as Cl/OH substitution. Hence, this work found important PPs that were not identified before. The work could extend to synthesizing transformation product standards and as a reference for the investigation of FLP and its transformation product residues in real samples such as foodstuff, environmental, and waste treatment effluents.

## Figures and Tables

**Table 1:** Photodegradation of FLP with retention times of the PPs (t_R_ in min), [*m/z*], the corresponding fragments on MS/MS, calculated *m*/*z*, measured *m*/*z*, deviation, and mechanism of formation.

Products	Molecular Formula	Retention Time (min)	Molecular Ion, [M + H]^+^	Product Ions (Q3), [M + H]^+^	Measured *m*/*z*, [M + H]^+^	δm/m, ppm	Mechanism of Formation from FLP
P1	C_14_H_8_F_6_N_2_O_2_	24.1	351.0563	335, 321, 291, 173, **164**, 145, 115	351.0555	2.2	−CH_2_=CH_2_, −Cl•, +HO•
P2 *	C_16_H_12_F_6_N_2_O	26.5	363.0927	385, 190, **173**, 145	363.0932	0.0	−Cl•, +H•
P3 *	C_16_H_12_F_6_N_2_O_2_	25.9	379.0881	417, 361, 208, **190**, 173, 152, 145	379.0880	−0.2	−Cl•, +HO•
P4	C_14_H_7_ClF_6_N_2_O_2_	26.3	385.0173	387, 407/409, **198**, 173, 162, 145	385.0169	−1.0	−CH_2_=CH_2_, +O
P5	C_16_H_10_F_6_N_2_O_3_	24.8 ^a^, 25.6 ^b^	393.0674	409, 427, 449, 375, 176, **188**	393.0682	2.0	−HCl, +2O
P6	C_16_H_10_F_6_N_2_O_4_	24.4	409.0618	431, 391, 377, 359, **220**, 202, 170, 150, 131	409.06899	3.8	−HCl, +3O
P7	C_16_H_9_ClF_6_N_2_O_3_	25.6 ^a^, 27.0 ^b^	427.0279	429, 449, 409, 353, 302, 206, 185, **173**, **145**	427.0256	−5.3	+2O, −2H•
P8 ^U^	-	30.2	533	**267**, 279, 289, 555, 249	-	-	-
P9	C_16_H_11_ClF_6_N_2_O_3_	22.3 ^a^, 24.1 ^b^, 26.7 ^c^, 28.5 ^b^	429.0435	431, 451, 411, 397, **240**, 222, 190, **173**, 145, 131, 115	429.0441	1.4	+2O
P10 *	C_16_H_10_F_6_N_2_O	31.8	361.0776	383, 343, **312**, 271, 190, 173, 145	361.0786	2.7	−HCl
P11	C_16_H_10_F_6_N_2_O_2_	28.2	377.0719	359, 345, 331, 176, **188**	377.0716	0.0	−HCl, +O
FLP	C_16_H_11_ClF_6_N_2_O	27.8/9	397.0535	419, 435, 208, 173, 145, 131, 115	397.0535	0.0	-

^a, b, and c^ on retention time: elution time of isomeric PPs, bold Q3: most abundant peak, * known before this work, U-unidentified yet, δm/m: relative mass deviation error (ppm).

## Supplementary Materials

The following are available online at http://www.mdpi.com/1420-3049/23/11/2940/s1, Figure S1: Chemical structure of fluopyram (FLP), Figure S2: MS/MS spectra of P10: lactam (a) and P3: hydroxyl-dechlorinated (b) FLP photodegradation products and their proposed fragmentation pattern, Figure S3. MS/MS spectra of P9: dihydroxyl (a) and P7: hydroxyl imide (b) FLP photodegradation products and their corresponding proposed fragmentation pattern, Figure S4: MS/MS spectra of P11: mono- (a) and P5: trihydroxyl (b) lactam FLP photodegradation products and their corresponding suggested fragmentation, Figure S5: MS/MS spectra of P4 (a), P1 (b), and P8 (c) formed by rearrangement and their proposed fragmentation mechanisms, Figure S6. EIC of P8 and m/z 267 with their respective Na+-adduct (a), EIC of selected TPs (b), kinetics of P8 and m/z 267 (c), and P4, P5, and P7 (d). The *y*-axis, ln(A_t_/A_0_), in (b) and (d) represents natural logarithm of peak area ratio of each PP after irradiated for time ‘t’ (A_t_) to before irradiated (A_0_) by considering a constant unity at t = 0.
