# Supplementary material for: New Photodegradation Products of the Fungicide Fluopyram: Structural Elucidation and Mechanism Identification
Source: Molecules. 2018 Nov 10;23(11):2940. doi: 10.3390/molecules23112940 (PMC6278505; doi:10.3390/molecules23112940)
Supplement: Supplementary file 1 [file molecules-23-02940-s001.pdf]

molecules

Article

New Photodegradation Products of the Fungicide Fluopyram: Structural Elucidation and Mechanism Identification

Tessema F. Mekonnen[1,2], Ulrich Panne[1,2] and Matthias Koch[1,*]

[1] Bundesanstalt für Materialforschung und –prüfung (BAM), Richard-Willstätter Str. 11, 12489 Berlin, Germany; tessema-fenta.mekonnen@bam.de; ulrich.panne@bam.de; matthias.koch@bam.de

[2] Humboldt-Universität zu Berlin, School of Analytical Sciences Adlershof (SALSA), Unter den Linden 6, 10099 Berlin, Germany; tessema-fenta.mekonnen@bam.de; ulrich.panne@bam.de

* Correspondence: matthias.Koch@bam.de; Tel.: +49 30 8104 1170

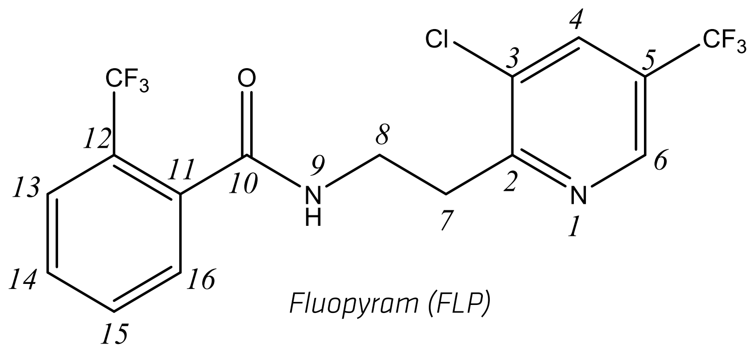

Figure S1. Chemical structure of fluopyram (FLP)

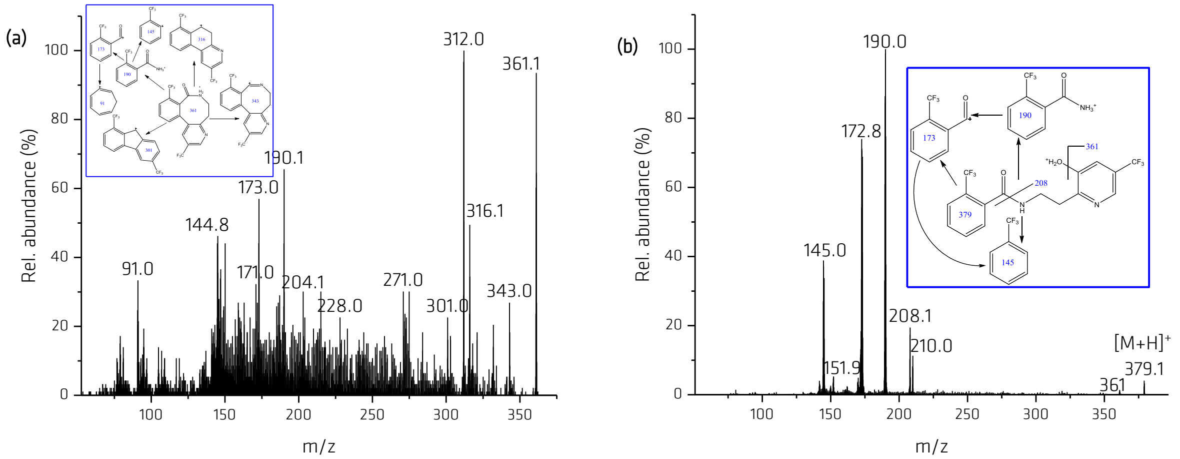

Figure S2. MS/MS spectra of P10: lactam (a) and P3: hydroxyl-dechlorinated (b) FLP photodegradation products and their proposed fragmentation pattern

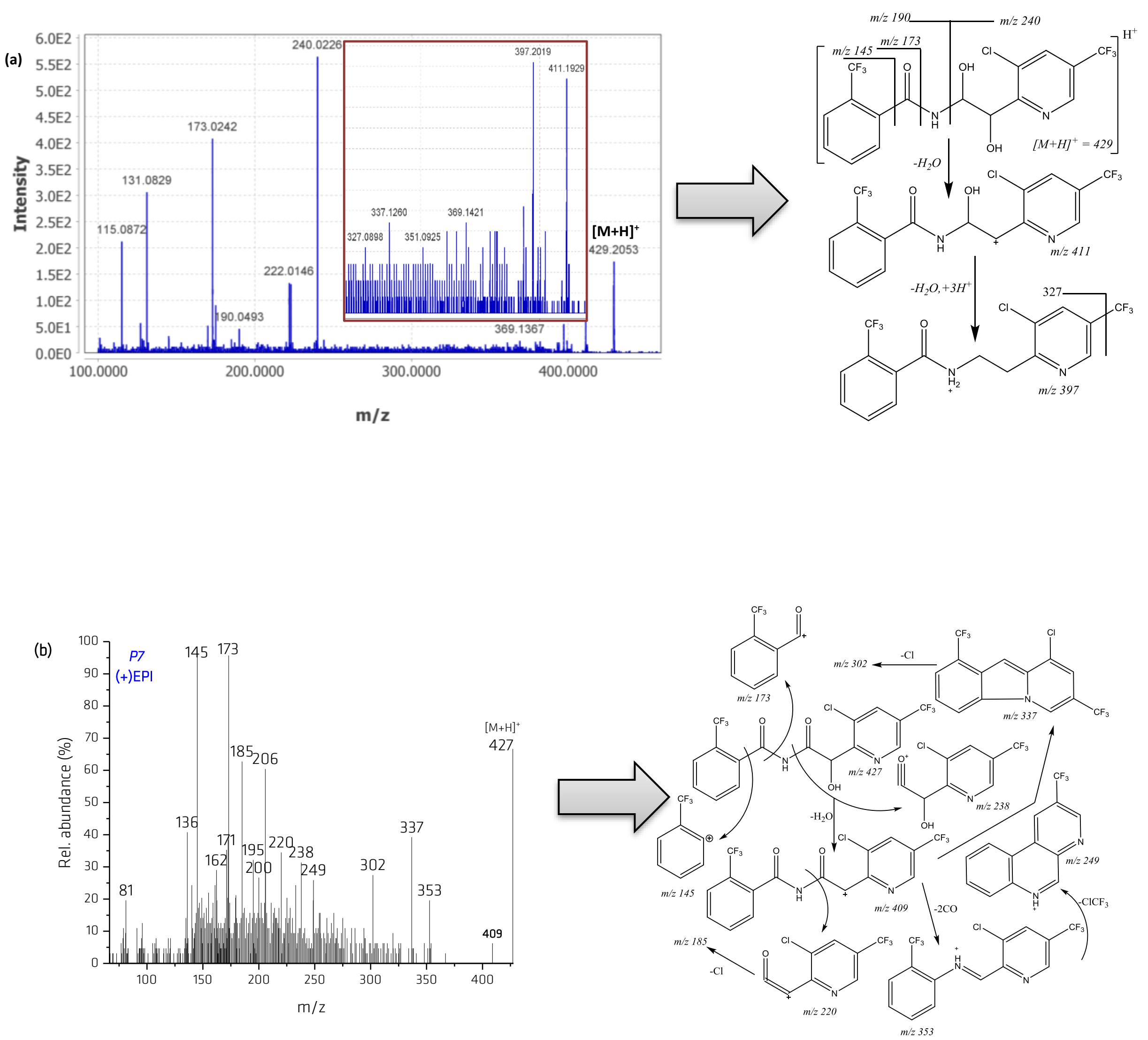

Figure S3. MS/MS spectra of P9: dihydroxyl (a) and P7: hydroxyl imide (b) FLP photodegradation products and their corresponding proposed fragmentation pattern

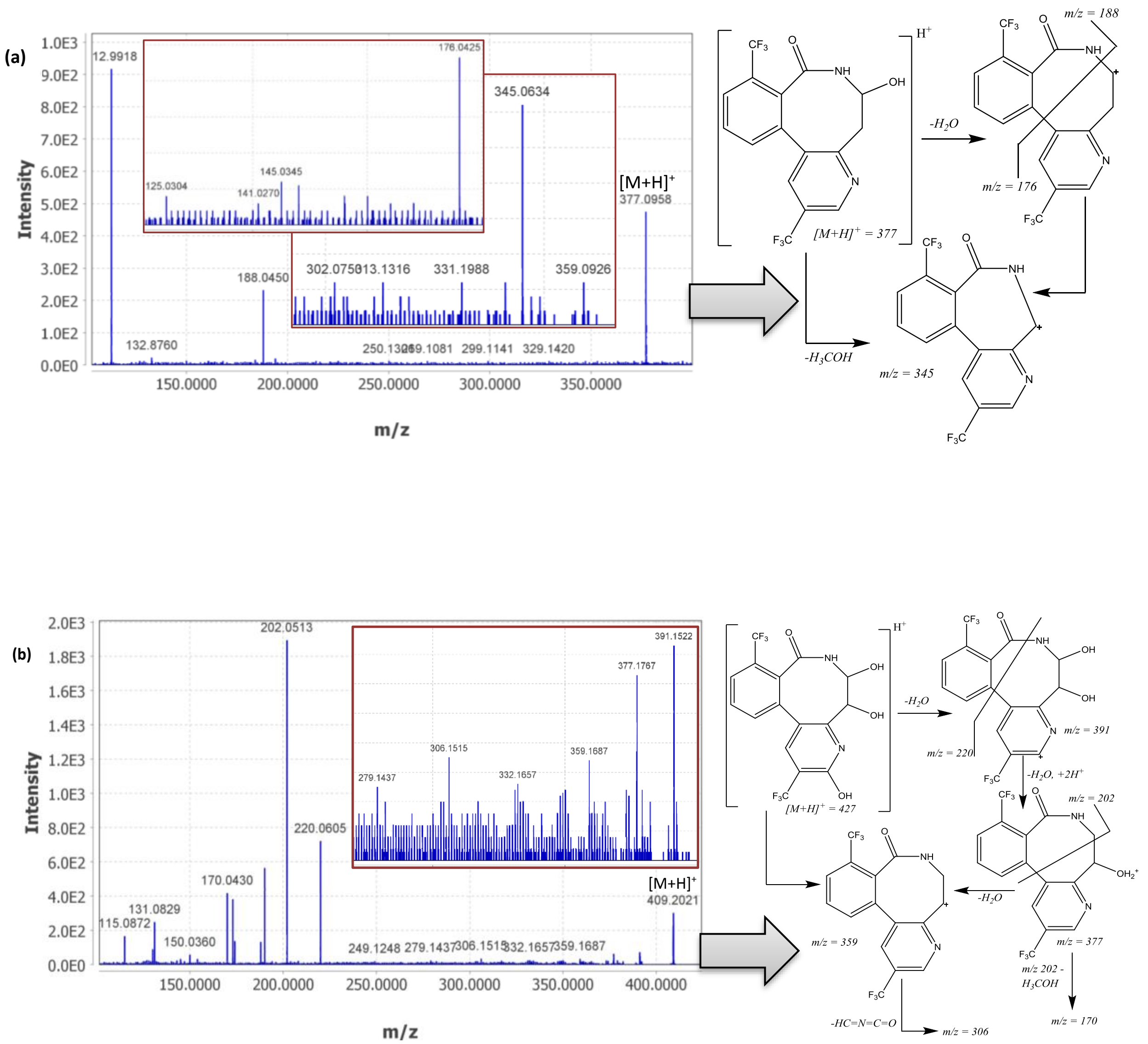

Figure S4. MS/MS spectra of P11: mono- (a) and P5: trihydroxyl (b) lactam FLP photodegradation products and their corresponding suggested fragmentation

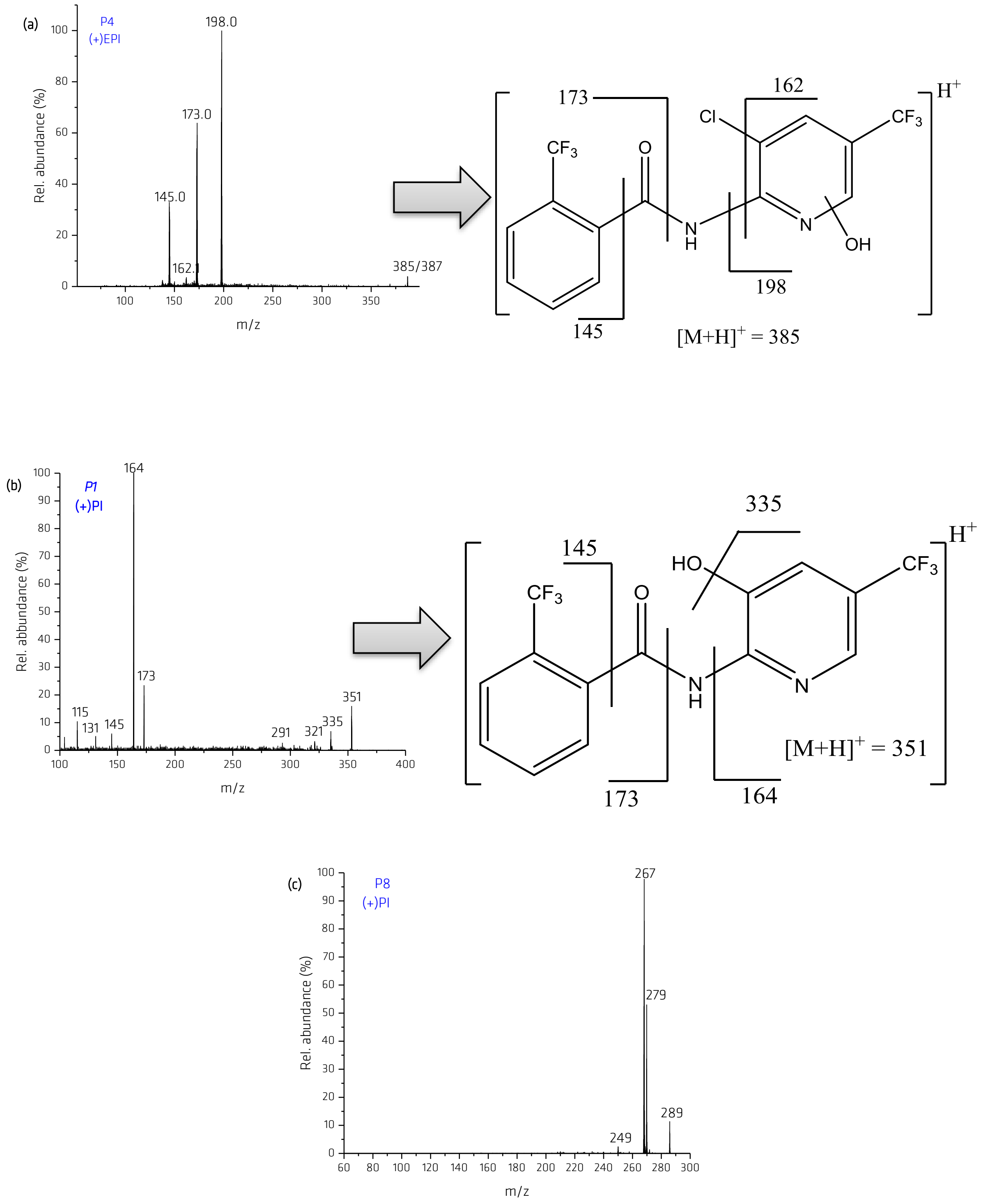

Figure S5. MS/MS spectra of P4 (a), P1 (b), and P8 (c) formed by rearrangement and their proposed fragmentation mechanisms

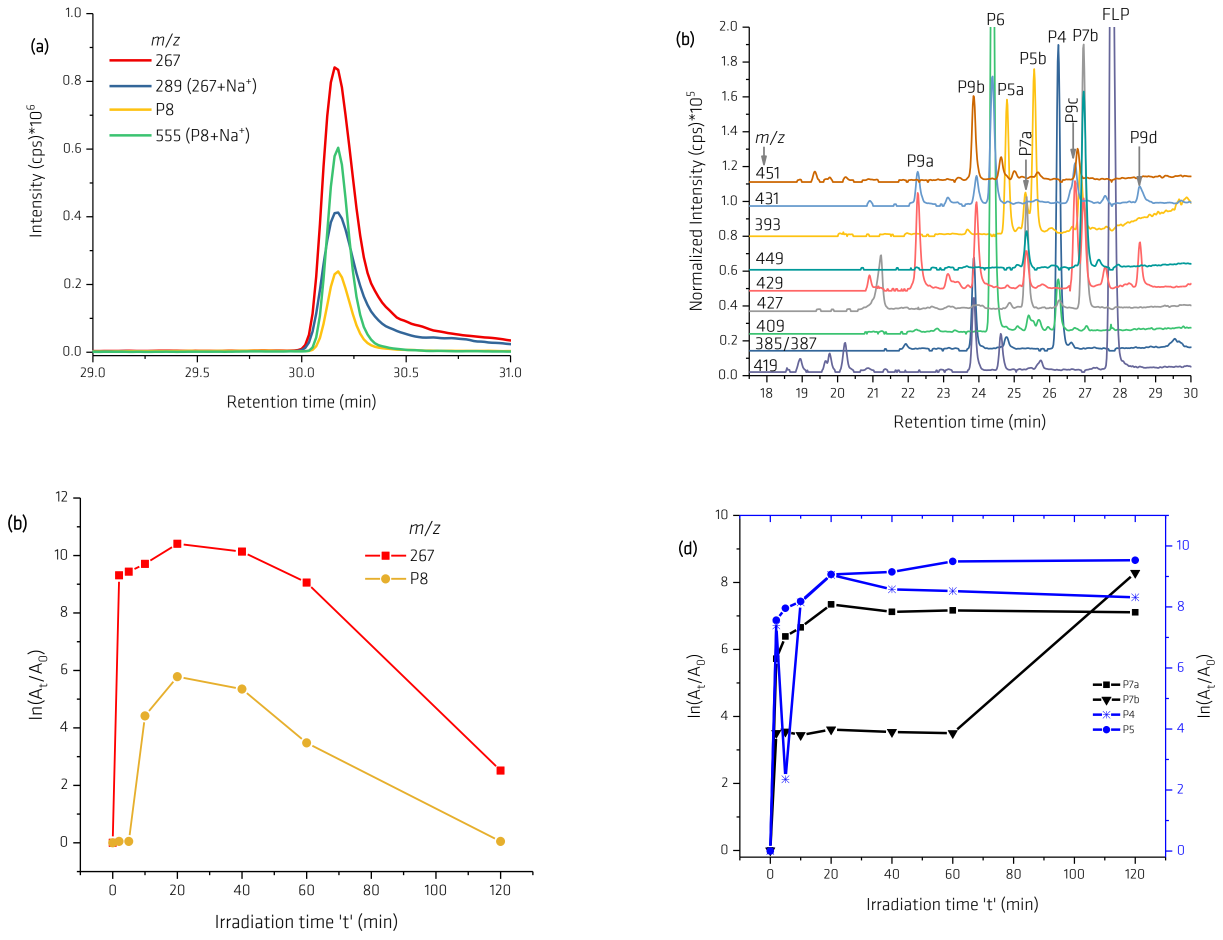

Figure S6. EIC of P8 and m/z 267 with their respective Na^+-adduct (a), EIC of selected TPs (b), kinetics of P8 and m/z 267 (c), and P4, P5, and P7 (d). The y-axis, $\ln(A_t/A_0)$, in (b) and (d) represents natural logarithm of peak area ratio of each PP after irradiated for time 't' (A_t) to before irradiated (A_0) by considering a constant unity at t = 0.
